# Supplementary material for: Stable centromere association of the yeast histone variant Cse4 requires its essential N-terminal domain
Source: EMBO J. 2025 Jan 14;44(5):1488–511. doi: 10.1038/s44318-024-00345-5 (PMC11876619; doi:10.1038/s44318-024-00345-5)
Supplement: Supplementary file 1 — Appendix [file 44318_2024_345_MOESM1_ESM.pdf]

Appendix to

Stable centromere association of the yeast histone variant Cse4 requires its essential N-terminal domain

Andrew R. Popchock, Sabrine Hedouin, Yizi Mao, Charles L. Asbury,
Andrew B. Stergachis and Sue Biggins

Table of Contents

p2 - Appendix Figure S1. Cse4 recruitment does not require the Cdc5 kinase.

p3 - Appendix Figure S2. Cse4^{S40D} regulates Scm3 but not Okp1/Ame1 recruitment.

p4 - Appendix Figure S3. Depletion of Okp1 reduces Cse4 centromeric recruitment *in vivo*.

p5 - Appendix Table S1: Related to Figures 1-7. List of *S. cerevisiae* strains used in this study

p9 - Appendix Table S2: Supplemental Table S2: Related to Figures 1-7. Plasmids used to generate *S. cerevisiae* strains, CEN DNA template generation and recombinant protein expression

p10 - Appendix Table S3: Related to Figures 1-6. DNA oligonucleotides used in this study for *S. cerevisiae* strain construction and CEN DNA template sequence generation

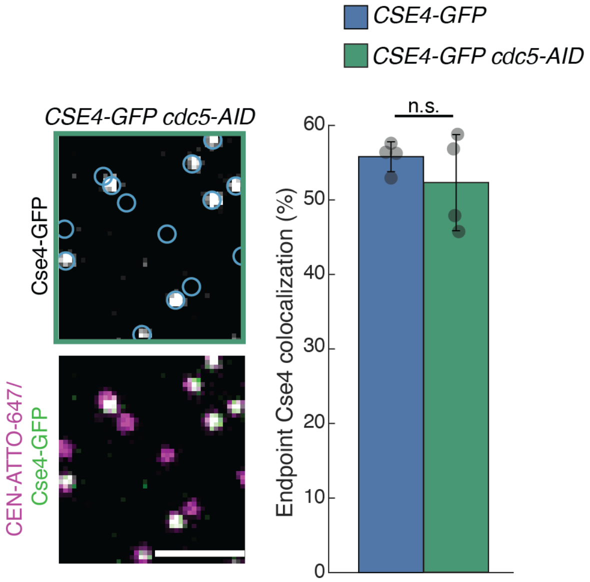

Appendix Figure S1. Cse4 recruitment depends on the Ipl1 kinase but not the Cdc5 kinase.

Example images of TIRFM endpoint colocalization assays. Top panels show visualized Cse4-GFP on CEN DNA in *CSE4-GFP cdc5-AID* (SBY23926) extracts (top panel) with colocalization shown in relation to identified CEN DNA in blue circles. Bottom panels show overlay of CEN DNA channel (magenta) with Cse4-GFP (green), scale bars 2 µm. Graph indicates quantification of Cse4-GFP endpoint colocalization with CEN DNA in extracts from *CSE4-GFP* or *CSE4-GFP ccdc5-AID* genetic backgrounds (56 ± 2.0%, 52 ± 6.5%, avg ± s.d. n = 4 experiments, each examining ~ 1,000 DNA molecules from different extracts, n.s. indicates two-tailed *P*-value of 0.4).

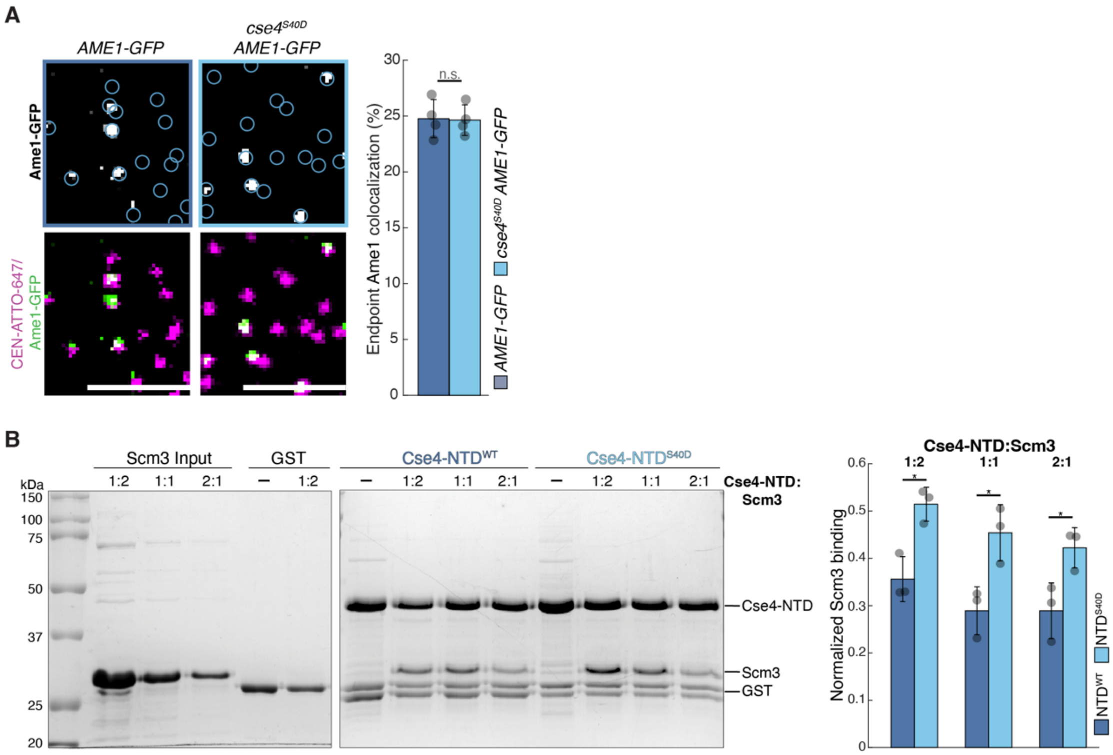

Appendix Figure S2. Cse4^{S40D} regulates Scm3 but not Okp1/Ame1 recruitment. A. Example images of TIRFM endpoint colocalization assays. Top panels show visualized Ame1-GFP on CEN DNA in WT (SBY22273) extracts (top-left panel) or *cse4^{S40D}* (SBY22403) extracts (top-right panel) with colocalization shown in relation to identified CEN DNA in blue circles. Bottom panels show overlay of CEN DNA channel (magenta) with Ame1-GFP (green), scale bars 3 µm. Graph indicates quantification of Ame1-GFP endpoint colocalization with CEN DNA in extracts from *AME1-GFP* or *cse4^{S40D} AME1-GFP* genetic backgrounds (25 ± 1.7%, 25 ± 1.4%, avg ± s.d. n = 4 experiments, each examining ~ 1,000 DNA molecules from different extracts, n.s. indicates two-tailed *P*-value of .9).

B. SDS-PAGE of GST pulldown assays (left) of immobilized Cse4-NTD^{WT} and Cse4-NTD^{S40D} to test binding of recombinant Scm3 at varying Scm3 concentrations. Quantification of Scm3 binding in pulldown assays (right) normalized to Cse4 levels for NTD^{WT} (0.36 ± 0.05, 0.29 ± 0.05, 0.29 ± 0.06) and the NTD^{S40D} mutant (0.51 ± 0.04, 0.45 ± 0.06, 0.42 ± 0.04) at varying input concentrations (1:2, 1:1, and 1:2 Cse4-NTD: Scm3 respectively, experiment was repeated three times to generate averages, * indicates two-tailed *P*-value of 0.01 for 1:2, 0.02 for 1:1 and 0.03 for 2).

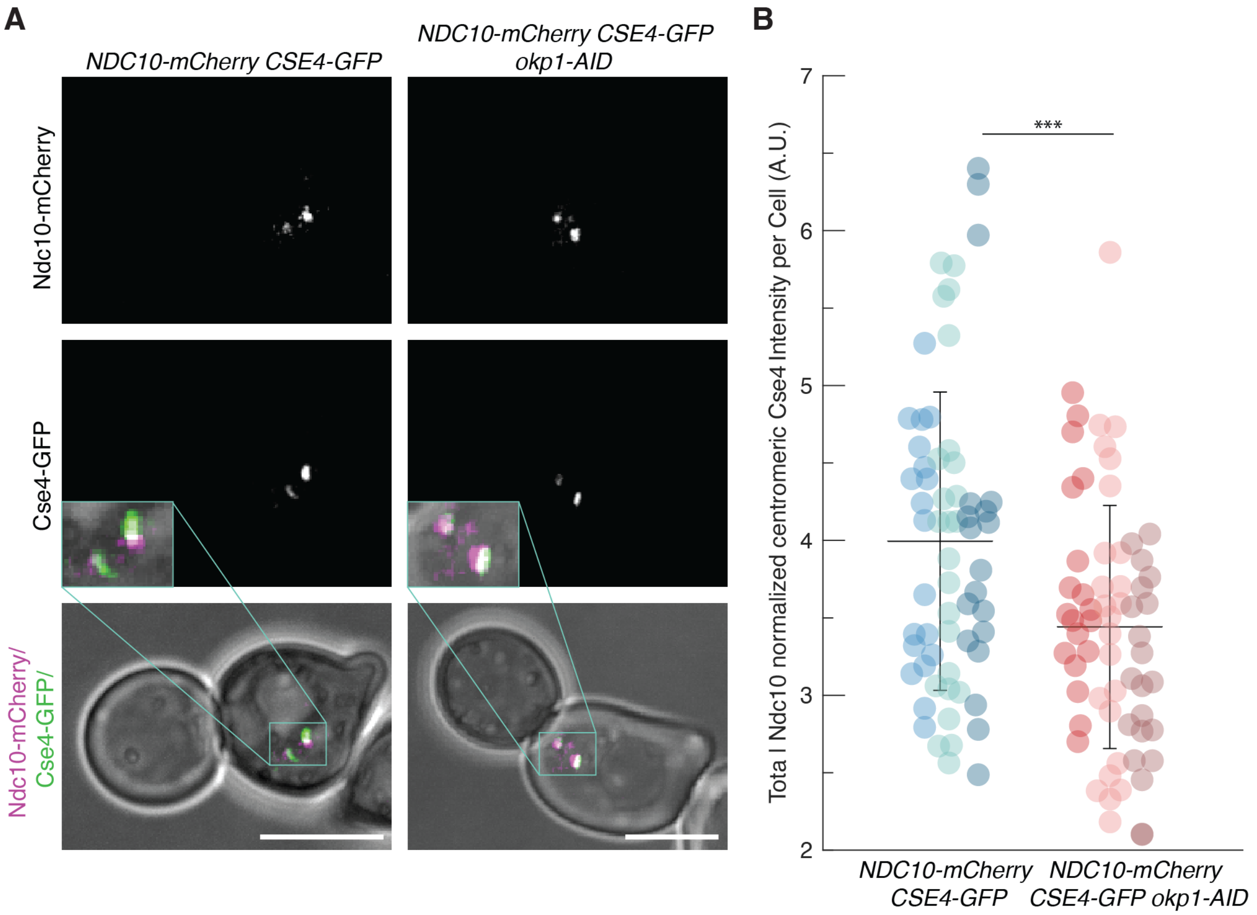

Appendix Figure S3. Depletion of Okp1 reduces Cse4 centromeric recruitment *in vivo*.

A. Example fluorescence microscopy images of *CSE4-GFP NDC10-mCherry* (SBY21974 - left) and *CSE4-GFP NDC10-mCherry okp1-AID* (SBY23022 - right) cells showing visualized Ndc10-mCherry (top panels), Cse4-GFP (middle panels) and overlay of Ndc10-mCherry (magenta) and Cse4-GFP (green) on plane polarized illumination of cell. Expanded region around kinetochores highlighted (middle panel inset). Scale bars 5 µm.

B. Graph indicates quantification of normalized centromere-associated Cse4-GFP intensity per cell of *CSE4-GFP NDC10-mCherry* (left) and *CSE4*-GFP *NDC10-mCherry okp1-AID* (right) cells (4.0 ± 1.0%, 3.4 ± 0.8%, avg ± s.d. n = 3 experiments, each examining ~ 25 cells). * Indicates significant difference as determined by t-Test (*P*-value of 5.6E-4). Each spot represents calculated intensity for one cell, different colors indicate biological replicates.

Appendix Table S1: Related to Figures 1-7. List of *S. cerevisiae* strains used in this study.

Strain	Genotype
	All strains derived from W303
SBY3	*MAT***a** *ura3-1 leu2,3-112 his3-11 trp1-1 ade2-1 LYS2 can1-100 bar1-1*
SBY4	*MATα ura3-1 leu2,3-112 his3-11 trp1-1 ade2-1 LYS2 can1-100 bar1-1*
SBY291	*MAT***a** *ura3-1 leu2,3-112 his3-11 trp1-1 ade2-1 LYS2 can1-100 bar1-1 mad1^HIS3*
SBY8315	*MAT***a** *ura3-1 leu2,3-112 his3-11 trp1-1 ade2-1 can1-100 bar1 NDC10-mCherry:HPH*
SBY15124	*MAT***a** *ura3-1 leu2,3-112 his3-11::his3::pGPD1-OsTIR1:HIS3 trp1-1 ade2-1 can1-100 bar1 okp1-3V5-IAA7:NatMX6*
SBY17186	*MAT***a** *ura3-1 leu2,3-112 his3-11::his3::pGPD1-OsTIR1:HIS3 trp1-1 ade2-1 can1-100 bar1 ipl1-3HA-IAA17:KAN*
SBY20017	*MATα ura3-1: leu2,3-112 his3-11 trp1-1 ade2-1 LYS2+ can1-100 bar1 ^cse4:KAN::cse4-S40D-XbaI(GFP):URA3*
SBY20019	*MATα ura3-1: leu2,3-112 his3-11 trp1-1 ade2-1 LYS2 can1-100 bar1 ^cse4:KAN::cse4-S40A-XbaI(GFP):URA3*
SBY20021	*MATα ura3-1: leu2,3-112 his3-11:: pGPD1-OsTIR1:HIS3 trp1-1 ade2-1 LYS2 can1-100 bar1 ^cse4:KAN::cse4-S40D-XbaI(GFP):URA3 ipl1-3HA-IAA17:KAN*
SBY20038	*MATα ura3-1: cse4-XbaI(GFP):URA3 leu2,3-112 his3-11:: pGPD1-OsTIR1:HIS3 trp1-1 ade2-1 LYS2 can1-100 bar1 ^cse4:KAN ^ctf19:Kan*
SBY20348	*MATα ura3-1 leu2,3-112 his3-11: pGPD1-OsTIR1:HIS3 trp1-1 ade2-1 LYS2 can1-100 bar1 ^cse4:Kan::cse4-S40D-XbaI(GFP):URA3 OKP1-3V5-IAA7:KanMX6*

SBY21863	*MATα ura3-1: leu2,3-112 his3-11 trp1-1 ade2-1 LYS2 can1-100 bar1 ^cse4:KAN::CSE4-XbaI(GFP):URA3*
SBY21972	*MATα ura3-1: leu2,3-112 his3-11:: pGPD1-OsTIR1:HIS3 trp1-1 ade2-1 LYS2 can1-100 bar1 ^cse4:KAN::CSE4-XbaI(GFP):URA3 ipl1-3HA-IAA17:KAN*
SBY21973	*MATα ura3-1: leu2,3-112 his3-11 trp1-1 ade2-1 LYS2 can1-100 bar1 ^cse4:KAN::CSE4-XbaI(GFP):URA3 Ndc10-mCherry:HPH*
SBY21974	*MAT***a** *ura3-1: leu2,3-112 his3-11 trp1-1 ade2-1 LYS2 can1-100 bar1 ^cse4:KAN::CSE4-XbaI(GFP):URA3 Ndc10-mCherry:HPH*
SBY22244	*MATα ura3-1: leu2,3-112 his3-11 trp1-1 ade2-1 LYS2 can1-100 bar1 ^cse4:KAN::CSE4-XbaI(GFP):URA3 Ame1-mKate2:HISMX6*
SBY22256	*MATα ura3-1: leu2,3-112 his3-11 trp1-1 ade2-1 LYS2 can1-100 bar1 ^cse4:KAN::CSE4-XbaI(GFP):URA3 Scm3-mKate2:HPH*
SBY22258	*MATα ura3-1: leu2,3-112 his3-11 trp1-1 ade2-1 LYS2 can1-100 bar1 ^cse4:KAN::CSE4-S40D-XbaI(GFP):URA3 Scm3-mKate2:HPH*
SBY22273	*MATα ura3-1: leu2,3-112 his3-11:: pGAL-CSE4-XbaI(GFP):LEU2 trp1-1 ade2-1 LYS2+ can1-100 bar1*
SBY22372	*MATα ura3-1: leu2,3-112 his3-11 trp1-1 ade2-1 LYS2 can1-100 bar1 ^cse4:KAN::CSE4-S40A-XbaI(GFP):URA3 Scm3-mKate2:HPH*
SBY22401	*MATα ura3-1: leu2,3-112 his3-11 trp1-1 ade2-1 LYS2 can1-100 bar1 cse4-S40D:NAT*
SBY22403	*MATα ura3-1: leu2,3-112 his3-11 trp1-1 ade2-1 LYS2 can1-100 bar1 cse4-S40D:NAT AME1-GFP:KanMX6*
SBY22405	*MATα ura3-1: leu2,3-112 his3-11 trp1-1 ade2-1 LYS2 can1-100 bar1 cse4-S40A:NAT*

SBY22431	*MAT**a** ura3-1 leu2,3-112 his3-11::his3::pGPD1-OsTIR1:HIS3 trp1-1 ade2-1 can1-100 bar1 ipl1-3HA-IAA17:KAN cse4-S40D:NAT*
SBY22656	*MAT**a** ura3-1 leu2-3,112::pADH1-OsTIR1:LEU2 his3-11 trp1-1 ade2-1 can1-100 bar1 cse4-3V5-IAA7-KanMX6*
SBY22720	*MAT**α** ura3-1: leu2,3-112 his3-11 trp1-1 ade2-1 LYS2 can1-100 bar1 ^cse4:KAN::CSE4-XbaI(GFP):URA3 Ame1-GFP:KanMX6*
SBY22803	*MAT**α** ura3-1: leu2,3-112 his3-11:: pGAL-cse4-^END-XbaI(GFP):LEU2 trp1-1 ade2-1 LYS2 can1-100 bar1*
SBY22811	*MAT**α** ura3-1 leu2,3-112 his3-11 trp1-1 ade2-1 LYS2 can1-100 bar1 ^cse4:Kan::cse4-L41A-XbaI(GFP):URA3*
SBY22914	*MAT**α** ura3-1 leu2,3-112 his3-11 trp1-1 ade2-1 LYS2 can1-100 bar1 ^cse4:Kan::cse4-S40D-L41A-XbaI(GFP):URA3*
SBY22929	*MAT**α** ura3-1 leu2,3-112 his3-11 trp1-1 ade2-1 LYS2 can1-100 bar1 ^cse4:Kan::cse4-S40D-L41A-XbaI(GFP):URA3 AME1-mKate2:HisMX6*
SBY22931	*MAT**α** ura3-1 leu2,3-112 his3-11 trp1-1 ade2-1 LYS2 can1-100 bar1 ^cse4:Kan::cse4-L41A-XbaI(GFP):URA3 AME1-mKate2:HisMX6*
SBY22987	*MAT**α** ura3-1 leu2,3-112 his3-11: pGPD1-OsTIR1:HIS3 trp1-1 ade2-1 LYS2 can1-100 bar1 ^cse4:Kan::cse4-XbaI(GFP):URA3 OKP1-3V5-IAA7:KanMX6*
SBY23022	*MAT**a** ura3-1: leu2,3-112 his3-1::pGPD1-OsTIR1:HIS3 1 trp1-1 ade2-1 LYS2 can1-100 bar1 ^cse4:KAN::CSE4-XbaI(GFP):URA3 NDC10-mCherry:HPH OKP1-3V5-IAA7:KanMX6*
SBY23099	*MAT**α** ura3-1 leu2,3-112 his3-11 trp1-1 ade2-1 LYS2 can1-100 bar1 NDC10-mCherry:Hph AME1-GFP:KanMX6*
SBY23101	*MAT**α** ura3-1 leu2,3-112 his3-11 trp1-1 ade2-1 LYS2 can1-100 bar1 ^cse4:Kan::cse4-L41A-XbaI(GFP):URA3 NDC10-mCherry:Hph*

SBY23105	*MATα ura3-1 leu2,3-112 his3-11 trp1-1 ade2-1 LYS2+ can1-100 bar1 ^cse4:Kan:CSE4-XbaI(GFP):URA3 ame1-I195Y-mKate2:HisMX6*
SBY23162	*MATα ura3-1 leu2,3-112 his3-11 trp1-1 ade2-1 LYS2+ can1-100 bar1 ^cse4:Kan:cse4-S40D-XbaI(GFP):URA3 ame1-I195Y-mKate2:HisMX6*
SBY23295	*MATα ura3-1 leu2,3-112 his3-11 trp1-1 ade2-1 LYS2 can1-100 bar1 ^cse4:Kan::cse4-L41A:URA NDC10-mCherry:Hph AME1-GFP:KanMX6*
SBY23237	*MATα ura3-1 leu2,3-112 his3-11 trp1-1 ade2-1 LYS2 can1-100 bar1 ^cse4:Kan::cse4-S40D-L41A:URA Ndc10-mCherry:Hph AME1-GFP:KanMX6*
SBY23474	*MATα ura3-1 leu2,3-112 his3-11 trp1-1 ade2-1 LYS2 can1-100 bar1 ^cse4:Kan::cse4-S40D-L41A-XbaI(GFP):URA3 NDC10-mCherry:Hph*
SBY23926	*MATα ura3-1 leu2,3-112: pGPD1-OsTIR1:LEU his3-11 trp1-1 ade2-1 LYS2 can1-100 bar1 ^cse4:Kan::cse4-XbaI(GFP):URA3 Cdc5-3V5-IAA7:KanMx6*

Appendix Table S2: Related to Figures 1-7. Plasmids used to generate *S. cerevisiae* strains, CEN DNA template generation and recombinant protein expression.

Plasmid	Description	Source
pSB64	*13MYC, HIS3MX6*	Biggins Lab
pSB963	*WT CEN3, 8LacO, TRP1*	Biggins Lab
pSB1582	*mCherry, HPH*	Biggins Lab
pSB1617	*pCSE4-cse4(1-80)-GFP-(81-229), URA3* (integrating)	Biggins Lab
pSB2066	*3V5-IAA7, KanMX6*	Biggins Lab
pSB2273	*pGPD1-OsTIR1, HIS3* (integrating)	Biggins Lab
pSB3218	*URA-CEN-gRNA-Cas9 plasmid targeting Cse4*	Biggins Lab
pSB3220	*pCSE4-cse4-S40D-(1-80)-GFP-(81-229), URA3* (integrating)	Biggins Lab
pSB3221	*pCSE4-cse4-S40A-(1-80)-GFP-(81-229), URA3* (integrating)	Biggins Lab
pSB3249	*pGAL-cse4(1-80)-GFP-(81-229), LEU2* (integrating)	Biggins Lab
pSB3263	*pCSE4-cse4(1-80)-mCherry-(81-229), URA3* (integrating)	Biggins Lab
pSB3431	*mKAte2, HISMX6*	Biggins Lab
pSB3449	pET21b - Scm3-6xHIS	Biggins Lab
pSB3456	*pSCM3-SCM3-mKate2 HPH, LEU (integrating)*	Biggins Lab
pSB3479	pET21b - GST	Biggins Lab
pSB3480	pET21b - GST-Cse4(1-131)	Biggins Lab
pSB3481	pET21b - GST-Cse4(1-131)-S40D	Biggins Lab
pSB3487	pET21b - GST-Cse4(1-131)-L41D	Biggins Lab
pSB3488	pET21b - GST-Cse4(1-131)-L41A	Biggins Lab
pSB3497	*pCSE4-cse4-L41A-(1-80)-GFP-(81-229), URA3* (integrating)	Biggins Lab
pSB3498	pET21b - GST-Cse4(1-27)-(61-131)	Biggins Lab
pSB3512	*EGFP, Kan*	Biggins Lab
pSB3526	*pCSE4-cse4-S40D-L41A-(1-80)-GFP-(81-229), URA3* (integrating)	Biggins Lab
pSB3568	*pCSE4-cse4-L41A, URA3* (integrating)	Biggins Lab
pSB3569	*pCSE4-cse4-S40D-L41A, URA3* (integrating)	Biggins Lab
pSB3590	*pGAL-cse4((1-27)-(61-80))-GFP-(81-229), LEU2* (integrating)	Biggins Lab
pSB3591	*URA-CEN-gRNA-Cas9 plasmid targeting Ame1*	Biggins Lab
pSB3592	pETDuet - Ame1-6xHIS, Okp1	Biggins Lab

Appendix Table S3: Related to Figures 1-6. DNA oligonucleotides used in this study for *S. cerevisiae* strain construction and CEN DNA template sequence generation.

Primer	Sequence	Purpose
SB51	5' - GGAGGCATGACCATCAAAATTCATTTGATGGTCTGTTAGTATATCTATCTAACCGGATCC CCGG GTTAATTAA - 3'	5' primer to tag *NDC10*
SB52	5' - CATACATGTCGGTATCCCTATACGAAACAGTTTAAACTTCGAAGCTCCCTCAGAATTCG AGCTC GT TTAAAC - 3'	3' primer to tag *NDC10*
SB64	5' - CCGAAAAAGGGAAAAATCGGCTCCAGCCCTGAAGCACAAATATCACTATCGATGAATT CGAGC TCGTT - 3'	3' primer to delete *CSE4*
SB67	5' - CAGAAGAAGGACTGAATATAGAAAGAATACTAATATAACATAATCCGGATCCCCGGGTT AATTA A- 3'	5' primer to delete *CSE4*
SB348	5' - CAAGCGTTGCCCTAAATATCTTCTATACTACAATTCATTTTAATTCAATATCCGGATCCCC GGGTT AATTAA- 3'	5' primer to tag *IPL1* with pSB2066
SB349	5' - CCCTCCTATTTTTAGATGAATCTCTAAGATCTAAATGCCCTCAACTTAAATATGTCGATGA ATTCG AGCTCGTT - 3'	3' primer to tag *IPL1* with pSB2066
SB1713	5' - TTGTCCACTTGTTGCCACTGA- 3'	3' primer to tag *SCM3* with mKATE2 with pSB3456

SB3878	5' - biotin-GGTTCTGGTGGTTCTGGTGGTTCTGGTGAATTCAAACAACCGCC GGCTTCCACCA - 3'	250 bp CEN3 for bulk assembly assays
SB3879	5' - biotin- GGTTCTGGTGGTTCTGGTGGTTCTGGTGGAATTCATTGTTTGTGCACTTGCCTGCA - 3'	750 bp CEN3, C0N3, templates
SB3880	5' - ATCAGCGCCAAACAATATGGAA - 3'	250 bp CEN3 for bulk assembly assays
SB4549	5' - GCACCATGAGTCGCACCAAGATAAGACCGAAGAAGATATACACCGGATCCCCGGGTT AATT AA- 3'	5' primer to tag *OKP1* with pSB2066
SB4550	5' - CAAATATTTAGTTATATGCATCGTAATCGTAAACTCTGAAACAATGGATTATCGGAATTCG AG CTCGTTTAAAC- 3'	3' primer to tag *OKP1* with pSB2066
SB4919	5’ - CTTCTGAAAAAGATAAATAAAATTAATGAAAATCTTTCTAACGAATTACAACCAAGTCTA CGGATCCCCGGGTTAATTAA - 3’	5' primer to tag *AME1* with pSB3512
SB4920	5’ - GACCTTATAACACAACTTCCTTAGTATGGAAGTAATACATATATACATATATACATATATAG AATTC GAGCTCGTTTAAAC - 3’	3' primer to tag *AME1* with pSB3431, pSB3512
SB7872	5' - AGTACGAGGCCAAACTCTCGAAAAGGATATTACGAGATGCGGCCGCTCTAGAACTAGT GG- 3'	5' primer to tag *SCM3* with mKate2with pSB3456
SB7870	5' - ATTO647-ATGGTGTTTATGCAAAGAAACCA - 3'	750 bp CEN3, C0N3 templates
SB8153	5’ - TTAGTTCTGCTATTCAAAGTGATTCGAGTGGAAGATCACTCAGTAACGTCAACAGGCTTG CAG	Template to mutate *cse4-L41A*

	GAGAtCAACAATCTATTAACGATCGTGCGTTATCGGCATTGCAGAGAACAAGAGCGACA AAGAACCTGTTTCCAAGAAGAGAGGAAAGAAGACGTTATGAAAGCTCAAAAAGTGACC TAGATATCGAAACAGACTAC - 3’	
SB8762	5’ - TGATGATCAATTTAGGAAACTATTGTACAAATTAGATCTGCGACTCTTTCAAACGATATCA GACC AAATGACCAGAGACTTGAAAGACATATTAGATTATAACGTGTCGAATAATGAGCTCTGCT ACCAACTGAAGCAAGTACTTGCTCGAAAAGAAGATTTGAACCAGCAGATAATATCGGTA CGAAATGAAATTCAAG - 3'	Template to mutate *ame1-I195Y*
SB8765	5’ – ATAAAATTAATGAAAATCTTTCTAACGAATTACAACCAAGTCTACGACGGATCGGTGACG – 3’	5' primer to tag *AME1* with pSB3431, pSB3512
