# Supplementary material for: Stable centromere association of the yeast histone variant Cse4 requires its essential N-terminal domain
Source: EMBO J. 2025 Jan 14;44(5):1488–511. doi: 10.1038/s44318-024-00345-5 (PMC11876619; doi:10.1038/s44318-024-00345-5)
Supplement: Supplementary file 3 — Expanded View Figures [file 44318_2024_345_MOESM3_ESM.pdf]

Expanded View Figures

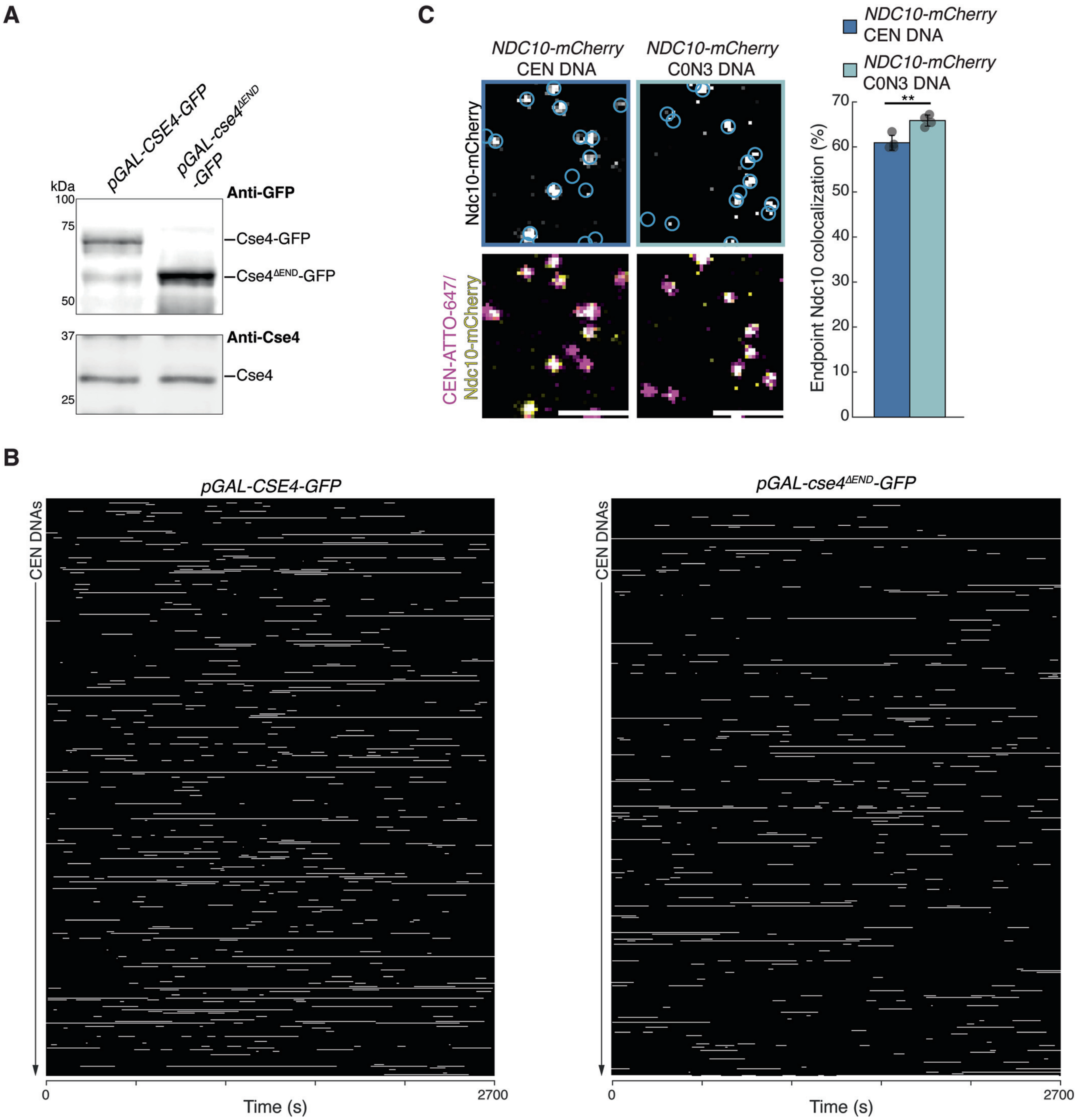

Figure EV1. Cse4 recruitment is impaired by loss of the END region or non-native centromeric DNA composition.

(**A**) Anti-GFP and anti-Cse4 immunoblots of whole-cell extract of *pGAL-CSE4-GFP* (left) and *pGAL-cse4$^{\Delta END}$-GFP* (right). (**B**) Example time-lapse TIRFM colocalization assay plots of residences of Cse4 on CEN DNA per imaging sequence in *pGAL-CSE4-GFP* (SBY22273) extracts (left) or *pGAL-cse4$^{\Delta END}$-GFP* (SBY22803) extracts (right). Each row represents one identified CEN DNA with all identified residences shown over entire imaging sequence (2700 s) for Cse4-GFP. (**C**) Example images of TIRFM endpoint colocalization assays. Top panels show visualized Ndc10-mCherry on CEN DNA (top-left panel), or on C0N3 DNA (top-right panel) in *NDC10-mCherry* (SBY8315) extracts with colocalization shown in relation to identified DNAs in blue circles. Bottom panels show overlay of CEN or C0N3 DNA channel (magenta) with Ndc10-mCherry (yellow), Scale bars 2 μm. Graph indicates quantification of Ndc10-mCherry endpoint colocalization with CEN DNA or C0N3 DNA (61 ± 1.7%, 66 ± 1.2%, avg ± s.d. $n = 4$ experiments, each examining ~1000 DNA molecules from different extracts, ** indicates significant difference with two-tailed P value of 0.003).

 © The Author(s)

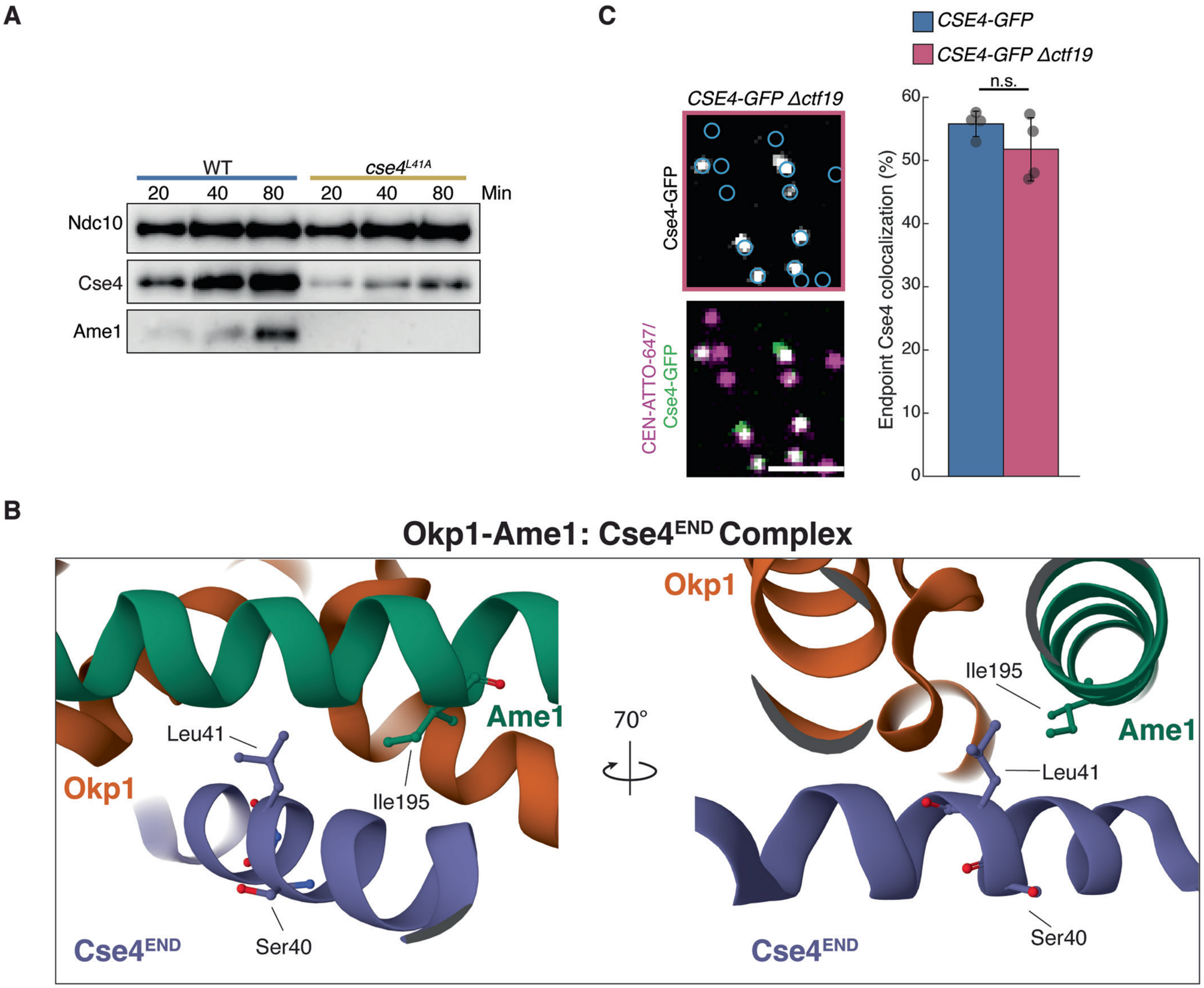

Figure EV2. Cse4 centromere recruitment is impaired by loss of the END binding to Okp1/Ame1 but not loss of the Ctf19 CCAN component.

(**A**) Immunoblots of bulk kinetochore assembly assays in WT (SBY3) (blue) or *cse4*^{L41A} (SBY22466) (orange) extracts on CEN DNA. Centromere DNA-bound proteins were analyzed by immunoblotting with the indicated antibodies. (**B**) Structure of Cse4^{END} (purple) in complex with Okp1/Ame1 (orange and green), highlighting hydrophobically packed residues Ile195 of Ame1 and Leu41 of Cse4 as well as Cse4 Ser40. Image of 8T0P adapted from (Deng et al, 2023) and created with Mol* (Sehnal et al, 2021). (**C**) Example images of TIRFM endpoint colocalization assays. Top panels show visualized Cse4-GFP on CEN DNA in CSE4-GFP *Δctf19* (SBY20038) extracts (top panel) with colocalization shown in relation to identified CEN DNA in blue circles. Bottom panels show overlay of CEN DNA channel (magenta) with Cse4-GFP (green), scale bars 2 µm. Graph indicates quantification of Cse4-GFP endpoint colocalization with CEN DNA in extracts from *CSE4-GFP* or *Cse4-GFP Δctf19* genetic backgrounds (56 ± 2.0%, 52 ± 5.0%, avg ± s.d. $n = 4$ experiments, each examining ~1000 DNA molecules from different extracts, n.s. indicates two-tailed P value of 0.2).

© The Author(s)

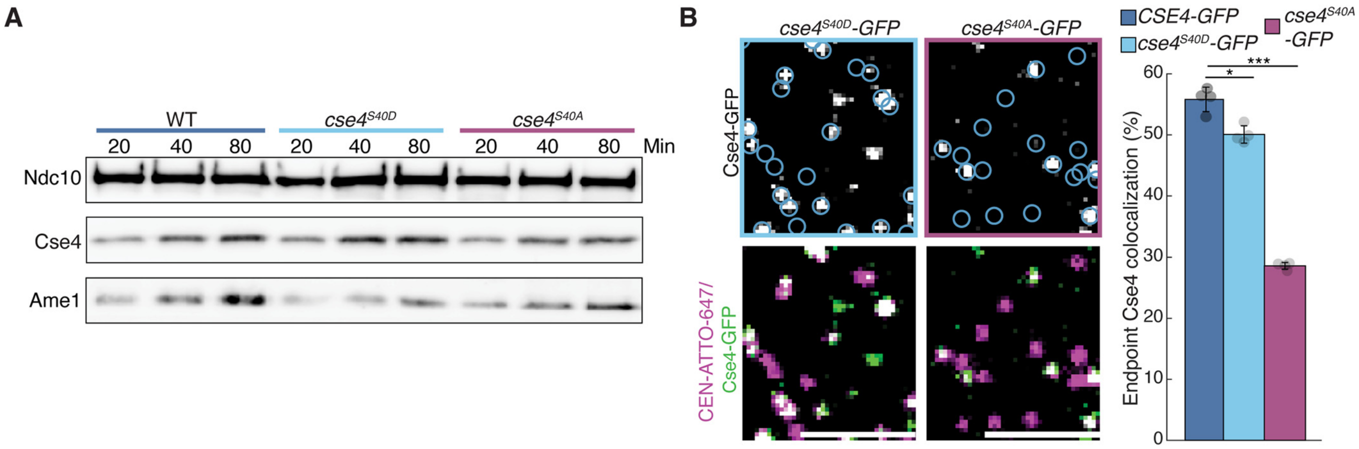

Figure EV3. Phosphorylation of the Cse4 END at S40 regulates Cse4 levels during kinetochore assembly.

(**A**) Bulk kinetochore assembly assays on centromeric DNA in WT (SBY4) (left), *cse4*^{S40D} (SBY22401) (middle), or *cse4*^{S40A} (SBY22405) (right) cell extracts. DNA-bound proteins were analyzed by immunoblotting with the indicated antibodies. (**B**) Example images of TIRFM endpoint colocalization assays. Top panels show visualized Cse4-GFP in extracts containing *cse4*^{S40D}*-GFP* (SBY20017) (top-left panel) or *cse4*^{S40A}*-GFP* (SBY20019) (top-right panel) on CEN DNA with colocalization shown in relation to identified CEN DNA in blue circles. Bottom panels show overlay of CEN DNA channel (magenta) with Cse4-GFP (green), Scale bars 3 μm. Graph indicates quantification of endpoint colocalization with CEN DNA of Cse4-GFP in extracts containing *cse4*^{S40D}*-GFP* or *cse4*^{S40A}*-GFP* (50 ± 1.4%, 29 ± 0.5% respectively, avg ± s.d. $n = 4$ experiments, each examining ~1000 DNA molecules from different extracts, * indicates significant difference between *CSE4-GFP* and *cse4*^{S40D}*-GFP* with two-tailed *P* value of.006, *** indicates significant difference between *CSE4-GFP* and *cse4*^{S40A}*-GFP* with two-tailed *P* value of 1.2E-4).

 © The Author(s)

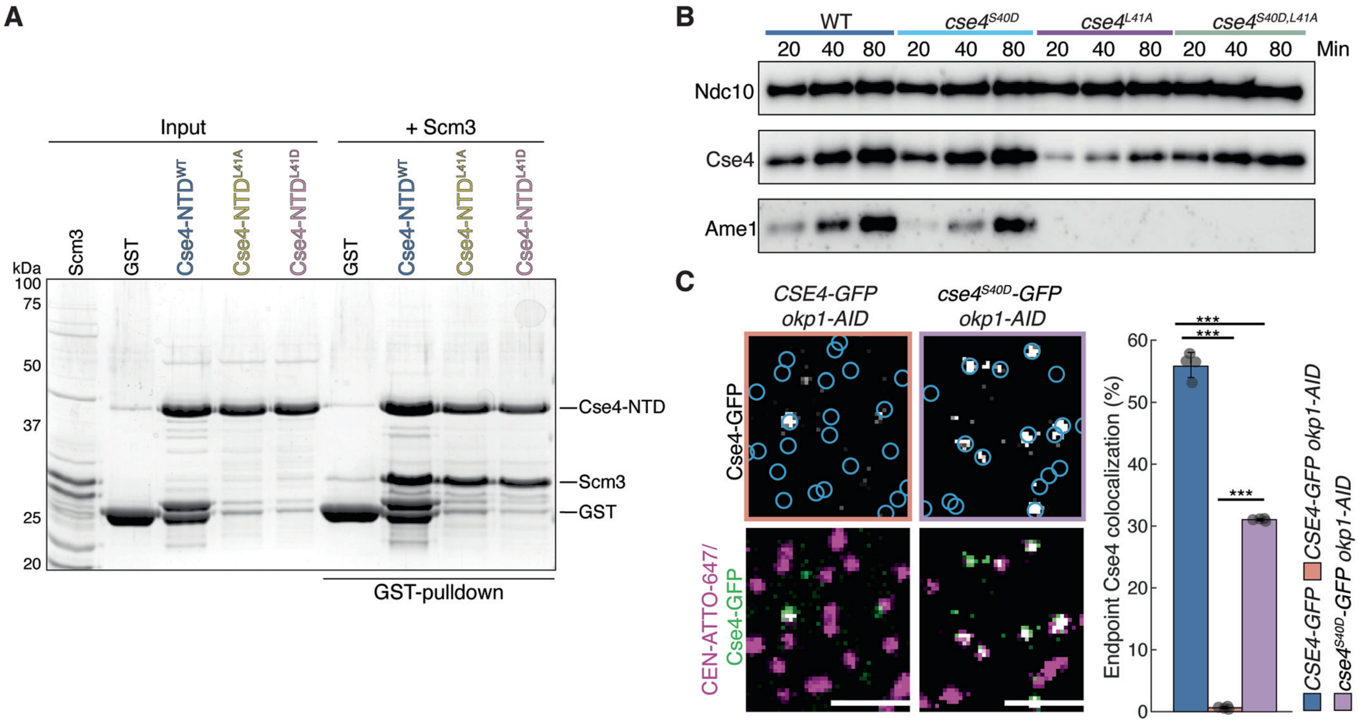

Figure EV4. Reduced Cse4 recruitment to centromeric DNA due to disruption of Okp1/Ame1 binding to Cse4 END in *cse4*^{L41A} mutant or by depletion of *OKP1* is rescued by Cse4^{S40D} mutant.

(**A**) SDS-PAGE of GST pull-down assays of immobilized GST-Cse4-NTD^{WT}, GST-Cse4-NTD^{L41A} and GST-Cse4-NTD^{L41D} to test binding of recombinant Scm3. (**B**) Bulk kinetochore assembly assays on CEN DNA in WT (SBY21863) extracts, *cse4*^{S40D} (SBY20017) extracts, *cse4*^{L41A} (SBY22811) extracts, or *cse4*^{S40D,L41A} (SBY22914) extracts. DNA-bound proteins were analyzed by immunoblotting with the indicated antibodies. (**C**) Example images of TIRFM endpoint colocalization assays. Top panels show visualized Cse4-GFP on CEN DNA in *CSE4-GFP okp1-AID* (SBY22987) extracts (top-left panel), or *cse4*^{S40D}*-GFP okp1-AID* (SBY20348) extracts (top-right panel) with colocalization shown in relation to identified CEN DNA in blue circles. Bottom panels show overlay of CEN DNA channel (magenta) with Cse4-GFP (green), scale bars 2 µm. Graph indicates quantification of Cse4-GFP endpoint colocalization with CEN DNA in extracts from *CSE4-GFP*, *CSE4-GFP okp1-AID*, or *cse4*^{S40D}*-GFP okp1-AID* genetic backgrounds (55 ± 0.5%, 1 ± 0.2%, or 31 ± 0.2%, avg ± s.d. $n = 4$ experiments, each examining ~1000 DNA molecules from different extracts, *** indicates significant difference between *CSE4-GFP* and *CSE4-GFP okp1-AID* with two-tailed *P* value of 1.3E-5, between *CSE4-GFP* and *cse4*^{S40D}*-GFP okp1-AID* with two-tailed *P* value of 1.5E-4, and between *cse4*^{S40D}*-GFP okp1-AID* and *CSE4-GFP okp1-AID* with two-tailed *P* value of 5.4E-13).

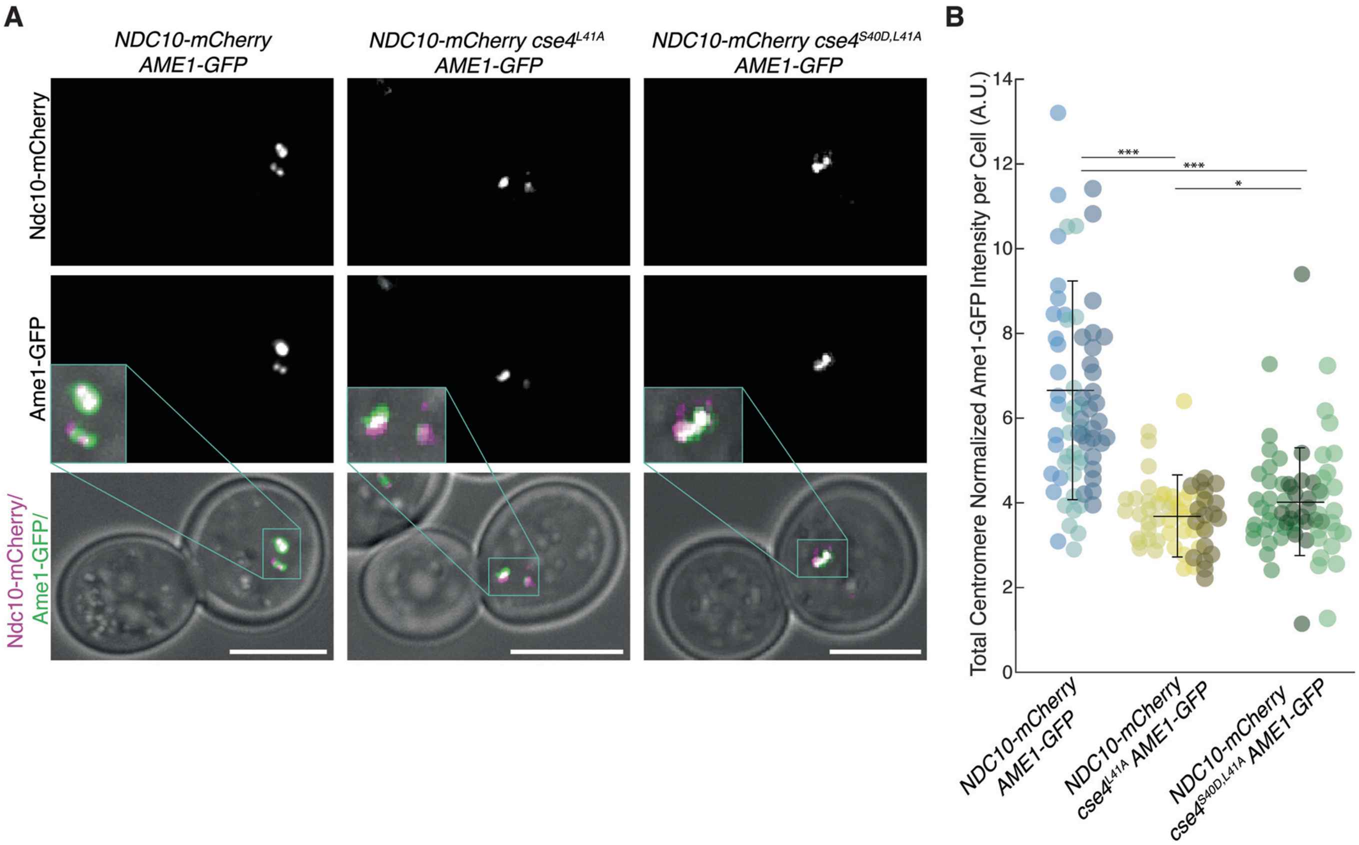

Figure EV5. Cse4^{S40D,L41A} mutant does not restore Okp1/Ame1 localization.

(**A**) Example fluorescence microscopy images of *NDC10-mCherry AME1-GFP* (SBY23099 - left), *NDC10-mCherry cse4^{L41A} AME1-GFP* (SBY23295 - middle) and *NDC10-mCherry cse4^{S40D,L41A} AME1-GFP* (SBY23237 - right) cells showing visualized Ndc10-mCherry (top panels), Ame1-GFP (middle panels) and overlay of Ndc10-mCherry (magenta) and Ame1-GFP (green) on plane-polarized illumination of cell. Expanded region around kinetochores highlighted (middle panel inset). Scale bars 5 µm. (**B**) Graph indicates quantification of Ndc10-mCherry normalized centromere-associated Ame1-GFP intensity per cell of *NDC10-mCherry AME1-GFP* (left), *NDC10-mCherry cse4^{L41A} AME1-GFP* (middle) and *NDC10-mCherry cse4^{S40D,L41A} AME1-GFP* (right) cells (6.7 ± 2.6%, 3.7 ± 1.0%, 4.0 ± 1.3%, avg ± s.d. $n = 3$ experiments, each examining ~ 25 cells). * Indicates significant difference as determined by *t* test (*NDC10-mCherry AME1-GFP*: *cse4^{L41A} AME1-GFP* P value of 2.7E-14, *NDC10-mCherry AME1-GFP* : *NDC10-mCherry cse4^{S40D,L41A} AME1-GFP* P value of 8.6E-12 and *NDC10-mCherry cse4^{L41A} AME1-GFP* : *cse4^{S40D,L41A} NDC10-mCherry AME1-GFP* P value of.09). Each spot represents calculated intensity for one cell, different colors indicate biological replicates.

 © The Author(s)
